# A role for TGFβ signalling in medium spiny neuron differentiation of human pluripotent stem cells

**DOI:** 10.1042/NS20200004

**Published:** 2020-05-06

**Authors:** Marija Fjodorova, Zoe Noakes, Meng Li

**Affiliations:** Neuroscience and Mental Health Research Institute, School of Medicine, School of Bioscience, Cardiff University, Cardiff CF24 4HQ, U.K.

**Keywords:** CTIP2, lateral ganglionic eminence, medium spiny neuron, neuronal differentiation, pluripotent stem cell, TGF-beta signaling

## Abstract

Activin A and other TGFβ family members have been shown to exhibit a certain degree of promiscuity between their family of receptors. We previously developed an efficient differentiation protocol using Activin A to obtain medium spiny neurons (MSNs) from human pluripotent stem cells (hPSCs). However, the mechanism underlying Activin A-induced MSN fate specification remains largely unknown. Here we begin to tease apart the different components of TGFβ pathways involved in MSN differentiation and demonstrate that Activin A acts exclusively via ALK4/5 receptors to induce MSN progenitor fate during differentiation. Moreover, we show that Alantolactone, an indirect activator of SMAD2/3 signalling, offers an alternative approach to differentiate hPSC-derived forebrain progenitors into MSNs. Further fine tuning of TGFβ pathway by inhibiting BMP signalling with LDN193189 achieves accelerated MSN fate specification. The present study therefore establishes an essential role for TGFβ signalling in human MSN differentiation and provides a fully defined and highly adaptable small molecule-based protocol to obtain MSNs from hPSCs.

## Introduction

Medium spiny neurons (MSNs) are the principal projection neurons of the striatum that receive synaptic inputs from both glutamatergic and dopaminergic afferents as part of the basal ganglia circuit. This arrangement is crucial for cognitive and motor functions, which include action selection, motor control, sequence learning, habit formation and more. Degeneration/dysfunction of MSNs has already been established as an early pathology in Huntington’s disease, where MSNs are the principle, albeit not the only, neurons affected [1]. There is also growing evidence for cortico- and midbrain-striatal systems’ imbalance as an etiological factor in a spectrum of neurodevelopmental and neuropsychiatric disorders such as autism and schizophrenia [2–5].

*In vitro*-derived human medium spiny neurons (MSNs) from pluripotent stem cells (hPSCs) are an invaluable tool for the study of striatal development and function, as well as a potential therapy in regenerative medicine. Previous works towards differentiating hPSCs into MSNs have focused on ventralising anterior neural progenitors produced from stromal cell co-culture, embryoid body or monolayer neural induction [6–9]. These groups acted on the principle that FOXG1^+^ cells in the developing telencephalon are exposed to opposing gradients of the ventralizing morphogen SHH and its repressor GLI3, which is expressed in dorsal tissues together with BMP and Wnt proteins [10]. Inducing SHH and inhibiting Wnt signalling in forebrain progenitors proved to be a successful strategy to generate pan-subpallial progenitors expressing FOXG1, OTX2, GSH2 and NKX2.1. However, CTIP2, FOXP1 and FOXP2 markers specific to lateral ganglionic eminence (LGE), the birthplace of MSNs, appeared quite late and only alongside DARPP32 expression after neuronal maturation [9]. Preferential induction of these markers was not apparent that suggests that these marker expressions are merely indicators of MSNs being born rather than a driving force for MSN induction. It is therefore likely that the MSNs generated using SHH and antagonists of Wnt result from the induction of pan-subpallial markers rather than a direct instructive effect towards LGE progenitors.

In contrast, we discovered that Activin A (Activin), a member of the TGFβ family of ligands, rapidly induces specific expression of MSN precursor markers in forebrain progenitors derived from hPSCs [11]. The TGFβ family of proteins, including TGFβ, Activins and BMPs, are involved in many aspects of cell proliferation, lineage determination, differentiation, motility, adhesion, and death [12]. Their intracellular effector proteins, the Smads, are phosphorylated upon receptor–ligand binding and translocate to the nucleus where they can activate gene transcription. Activins act through activin type II receptors A/B (ActR-IIA/B) and activin-like kinase receptors 4/5 (ALK4/5) to recruit Smad2/3. Phosphorylated Smad2 (pSmad2) was previously detected to co-localise with transcription factor DLX2 throughout the ganglionic eminences (GE) [13,14]. The two proteins were also confirmed to interact *in vivo* by immunoprecipitation, and pSmad2 was found to bind to the DLX2 gene enhancer by chromatin immunoprecipitation. In line with these findings, we observed robust induction of not only subpallial DLX2 and GSH2 expression but also of LGE-specific CTIP2 expression [11]. Moreover, we demonstrated in the same study that Activin acts independently of SHH and blockade of ALK4/5 receptors abolished any response to Activin, suggesting signalling mechanism via the ALK4/5-Smad2/3 pathway.

Use of small molecules for hPSC-differentiation offers many advantages over recombinant proteins – namely their increased stability and consistency in activity, as well as much lower usage cost. Alantolactone is a compound extracted from the roots of Inula helenium (Compositae), which grows in Europe and Asia, and was found to have anti-proliferative effects that have fuelled extensive research into a role in tumour suppression [15,16]. The mechanism by which Alantolactone suppresses tumours is shown to involve disruption of Cripto-1, an endogenous Activin antagonist, binding to the ActR-IIA [17].

Here, we investigated how fine tuning the TGFβ signalling to simultaneously induce Activin and inhibit BMP signalling affects MSN differentiation. Our study provides evidence that Alantolactone can replace Activin in inducing MSN fate from hPSCs. Thus, we provide a fully defined and highly adaptable small molecule-based MSN differentiation protocol.

## Methods

### Cell culture and MSN differentiation

MSNs were differentiated from the following hESC lines: H7 hESCs, H9 hESCs and HUES9 iCas9 hESCs. MSNs were obtained and maintained as described previously [11]. For inhibitor experiments, from 10DIV (days of *in vitro* differentiation) cells were treated with Activin alone (25 ng/ml) or plus the following inhibitors: SB431542 (10 µM), SB525334 (1 µM), LY2109761 (5 µM) or LDN193189 (100 nM). Concentrations for SB431542 and LDN193189 we chosen the same as in neural induction step of MSN differentiation protocol [11]. Concentrations for SB525334 and LY2109761 we chosen based on published literature [18,19]. In Alantolactone experiments, cells were supplemented with Alantolactone from 10DIV until 40DIV at the following concentrations: 0, 62.5, 125, 250, 500 nM.

### RT-PCR

Total mRNA was extracted from TRIzol lysates using the PureLink RNA mini kit (Ambion). RT-PCR was completed using 2 µg mRNA and the EvoScript kit (Roche). MesaGreen kit was used to perform qPCR using 200 pg mRNA/reaction. The following primers were used:

**Table:** 

Gene	Forward primer	Reverse primer
GAPDH	ACGACCCCTTCATTGACCTCAACT	ATATTTCTCGTGGTTCACACCCAT
β-ACTIN	TCACCACCACGGCCGAGCG	TCTCCTTCTGCATCCTGTCG
CTIP2	CTCCGAGCTCAGGAAAGTGTC	TCATCTTTACCTGCAATGTTCTCC
GSH2	TCACTAGCACGCAACTCCTG	TTTTCACCTGCTTCTCCGAC
DLX2	ACTACCCCTGGTACCACCAGAC	TCTGCTCTCAGTCTCTGGCGAGTTCTC
NKX2.1	CGCATCCAATCTCAAGGAAT	TGTGCCCAGAGTGAAGTTTG
PAX6	AATAACCTGCCTATGCAACCC	AACTTGAACTGGAACTGACACAC

### Immunocytochemistry

Cells were fixed for immunostaining in 3.7% PFA for 15 min. Cells were incubated with primary antibodies in blocking solution overnight at 4°C. Following three PBS-T washes, Alexa-Fluor secondary antibodies (Thermo Fisher Scientific) were added at 1:1000 for 1 h at ambient temperature in the dark. Cells were stained with DAPI at 1:1000 (Thermo Fisher Scientific). The following primary antibodies were used for the immunofluorescence studies: rat anti-CTIP2 (Abcam #ab18465, 1:500), rabbit anti-FOXP2 (Abcam #ab16046, 1:500), mouse anti-FOXP1 (Abcam #ab32010, 1:800), rabbit anti-GSH2 (Merck Millipore #ABN162, 1:500), mouse anti-ISL1 (Developmental Studies Hybridoma Bank #39.4D5, 1:500), rabbit anti-FOXG1 (Abcam # ab18259, 1:1000), rabbit anti-DARPP32 (Santa Cruz Biotechnology #sc-11365, 1:500). Cell fate markers were counted as a proportion of DAPI from at least five randomly selected fields/sample in ImageJ (imagej.net) blind to the experimental condition.

### Western blot

Equal amounts of proteins for each sample were separated on 4–12% Bolt Bis-Tris Plus gels (Thermo Fisher Scientific) and transferred via electro-blotting to a PVDF membrane (0.45 µm pore size, GE Healthcare). Membranes were incubated with primary antibodies overnight at 4°C followed by horseradish peroxidase-conjugated secondary antibodies (Abcam) for 1 h. The following primary antibodies were used for the Western blot studies: rabbit anti-pSmad2-Ser465/467 (Cell Signalling Technology #3101, 1:1000), rabbit anti-Smad2/3 (Cell Signalling Technology #3102, 1:1000), mouse anti-GAPDH (Abcam #8245, 1:10000). All blot images were quantified in ImageJ blind to the experimental condition.

### Statistical analysis

All quantified data were plotted in Prism 6 (GraphPad Software) and are reported as mean ± SEM with sample sizes for each test indicated in the figure legends. Data were pooled from either multiple hESC lines or independent experiments within the same line or a combination of both to acquire at least three independent observations per group. Box-and-whisker plots depict data for each condition: centre line – median,’+’ – mean, box limits – upper and lower quartiles, whiskers – 2.5 and 97.5 percentiles. Normal distribution of data were assessed with Kolmogorov–Smirnov test and homogeneity of variance was tested with Levene’s test. Statistical analysis was performed using two-tailed Student's *t*-test, one-/two-way ANOVA tests followed by Bonferroni’s correction for multiple comparisons if applicable or non-parametric Mann–Whitney test for not normally distributed data. Results were considered statistically significant at *P* < 0.05 and only significant results are reported on the graphs.

## Results

### Differential roles for TGFβ signalling in LGE fate induction in hPSCs

**Figure 1:**
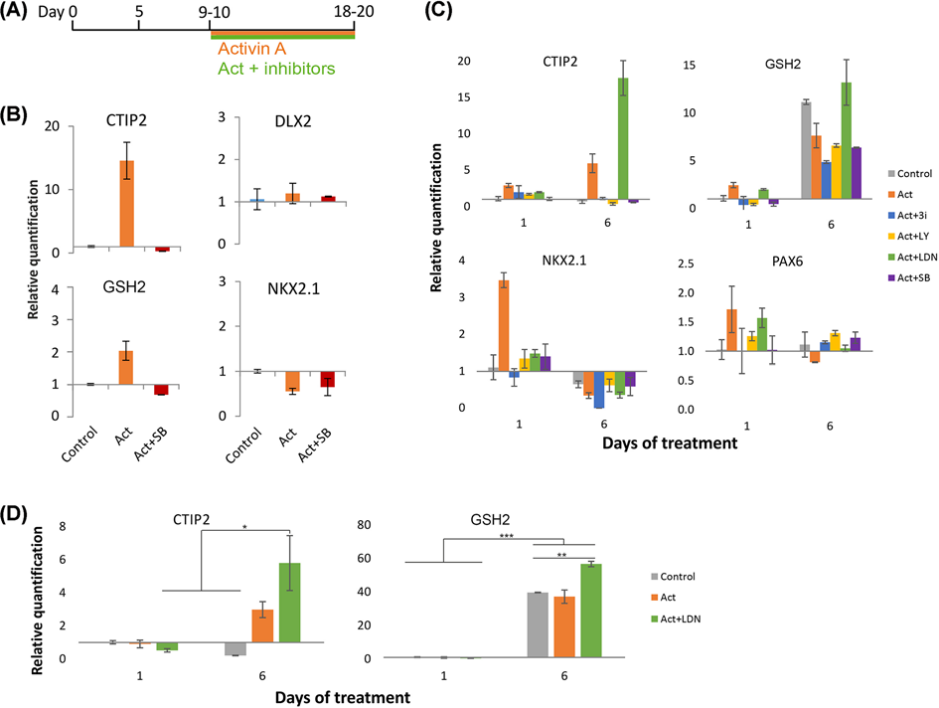
Differential roles for TGFβ signalling in LGE fate induction in hPSCs (**A**) Schematic timeline of MSN differentiation. (**B**) qPCR data from RNA extracted at 19DIV after treatment with Activin (25 ng/ml) alone or plus SB431542 (10 µM) as shown in panel (A). Data show mean RQ ± SEM relative to control (*n* = 6, 6 and 2, respectively). (**C**) qPCR data from RNA extracted 1 or 6 days after treatment that began at 10DIV. Cells were untreated (control) or treated with Activin (25 ng/ml), or Activin plus SB525334 (1 µM), LY2109761 (5 µM) or LDN193189 (100 nM). Data show mean RQ ± SEM relative to the 10DIV+1 control (*n* = 2 per group). (**D**) A repeat of the experiment in panel (**C**) with just control, Activin and Activin plus LDN conditions to confirm the LDN effects on *CTIP2* and *GSH2* up-regulation. Data show mean RQ ± SEM relative to the 10DIV+1 control (*n* = 3 per group; *P<0.05, **P<0.01, ***P<0.001; two-way ANOVA with post-hoc Bonferroni).

Activin and other TGFβ family members have been shown to exhibit a certain degree of promiscuity between their family of receptors [20]. In order to verify that Activin was acting via the ALK4/5 receptors to induce LGE gene up-regulation, Activin treatment of forebrain progenitors at 10 days *in vitro* (DIV) was supplemented with an ALK4/5 inhibitor, SB431542 (Figure 1A). At 19DIV, Activin-induced expression of *CTIP2* and *GSH2* was abolished in the presence of the inhibitor confirming receptor-specific activity (Figure 1B). Moreover, there was no change in *DLX2* and *NKX2.1* level in the Activin condition, further highlighting direct LGE fate specification by Activin versus alternative GE progenitor fates.

With the aim of teasing apart the different TGFβ pathways downstream of Activin induction in hPSC-derived forebrain progenitors, we performed a screen of three chemical inhibitors selective for different ALK receptors. SB525334 (SB5) is a dual ALK4/5 inhibitor and LY2109761 (LY) is a selective ALK5 inhibitor while both are inactive at ALK2/3/6. LDN193189 (LDN) is a selective ALK2/3/6 inhibitor that only affects BMP signalling. Cells were treated with Activin with or without inhibitors from 10DIV and then lysed for RNA extraction 1 or 6 days later (Figure 1A,C). Levels of mRNA from all conditions and time points were normalised to the control condition at 10DIV+1.

At 10DIV+1, Activin alone induced an increase in expression of *CTIP2, GSH2* and *NKX2.1* compared with the untreated control condition (Figure 1C). This effect was mostly abolished by all the inhibitors, with LDN consistently showing the least inhibition across all genes. At 10DIV+6, the level of *CTIP2* rose by 6-fold in the Activin condition, which was completely inhibited by SB5 and LY, the TGFβ/Activin pathway inhibitors. Interestingly, the addition of LDN to Activin caused an 18-fold increase in *CTIP2* mRNA levels, although this was also completely abolished by the further addition of both SB5 and LY. *GSH2* expression increased 11-fold in the control condition, whereas Activin alone only led to a 7.6-fold increase. Again, Activin plus LDN saw a modest 13.2-fold increase in *GSH2* compared with the control. *NKX2.1* mRNA level dropped to nearly undetectable levels in all conditions. *PAX6* expression was consistently lower than the subpallial markers and remained constant in all conditions across all time points.

The novel discovery from the Activin plus LDN condition was verified using a second qPCR experiment repeating the control, Activin and Activin+LDN conditions at the same timepoints (Figure 1D). The addition of LDN induced a significant increase in *CTIP2* mRNA compared to all conditions at 10DIV+1 and the control condition at 10DIV+6. Again, there was a large and significant up-regulation of *GSH2* in the control condition (39.6-fold), which was closely matched by the Activin treatment. Activin+LDN supplementation caused a further increase (56.7-fold), which was significantly different from both control and Activin alone.

### Inhibition of BMP signalling accelerates MSN differentiation

**Figure 2:**
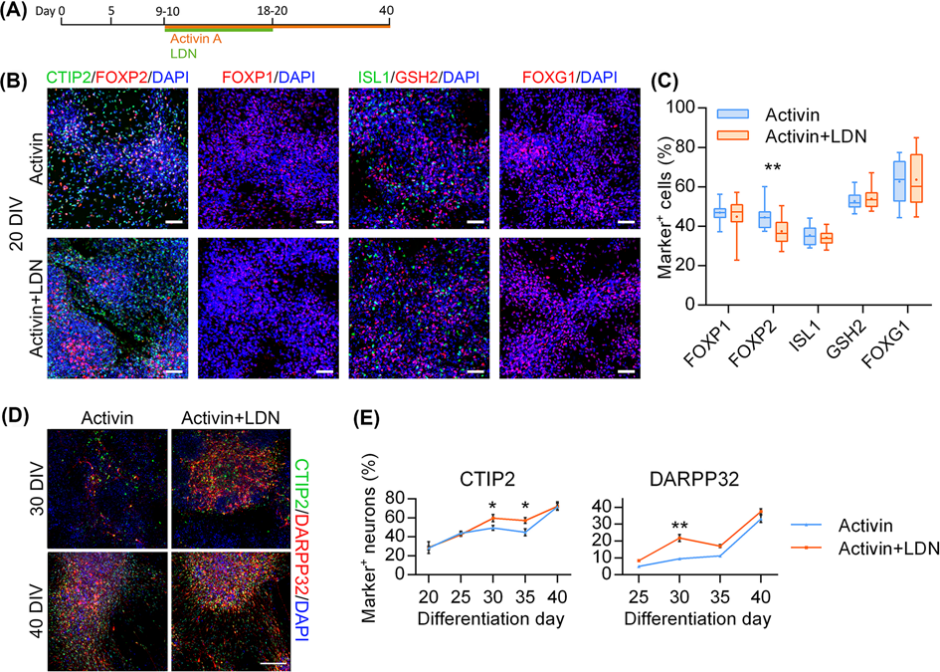
Inhibition of BMP signalling accelerates Activin effects (**A**) Schematic timeline of MSN differentiation. (**B** and **C**) Neuronal precursors expressing LGE and forebrain markers in Activin and Activin+LDN cultures at 20DIV labelled and quantified for FOXP1, FOXP2, ISL1, GSH2, and FOXG1 (*n* = 20 per group; **P<0.01; two-tailed Student’s *t*-test). Box-and-whisker plots depict data for each condition; scale bars: 50 µm. (**D** and **E**) MSNs in Activin and Activin+LDN cultures at 30DIV and 40DIV labelled and quantified for CTIP2 and DARPP32 (*n* = 10 per group; *P<0.05, **P<0.01; two-way ANOVA with post-hoc Bonferroni). Data are presented as mean ± SEM; scale bar: 50 µm.

The novel finding that BMP signalling blockade by LDN could enhance Activin-induced LGE gene up-regulation prompted further enquiry into its effects on protein expression at the MSN stage of differentiation. Similar to the previous experiment, differentiating neural progenitors were treated with Activin with or without LDN from 10DIV onwards. Activin supplementation in both conditions continued throughout the experiment, while addition of LDN was stopped at 20DIV based on a preliminary experiment (Figure 2A). At 20DIV, both Activin and Activin+LDN treatments produced comparatively high yields of neuronal precursors expressing LGE markers CTIP2, FOXP1, FOXP2, ISL1, GSH2 and forebrain marker FOXG1 (Figure 2B,C). Only FOXP2^+^ cell numbers were significantly higher in Activin condition compared with Activin+LDN.

The percentage of CTIP2^+^ neurons increased between 20DIV and 40DIV in all conditions, from approximately 30% to 70% (Figure 2D,E). Similarly, DARPP32^+^ neuron population increased in both conditions from less than 10% at 25DIV to up to 40% at 40DIV. Interestingly, there were significantly higher percentages of CTIP2^+^ neurons at 30DIV and 35DIV in Activin+LDN condition compared with Activin alone (Figure 2E). This effect was also present in the number of DARPP32^+^ neurons, which was more than doubled at 30DIV in Activin+LDN condition in contrast with Activin alone. However, by 40DIV the numbers of CTIP2^+^ and DARPP32^+^ neurons in the Activin condition reached a similar level to that of Activin+LDN condition. These results suggest that inhibition of BMP signalling accelerate differentiation of LGE-like progenitors into MSNs.

### Efficient induction of MSN fate by TGFβ small molecule agonist

**Figure 3:**
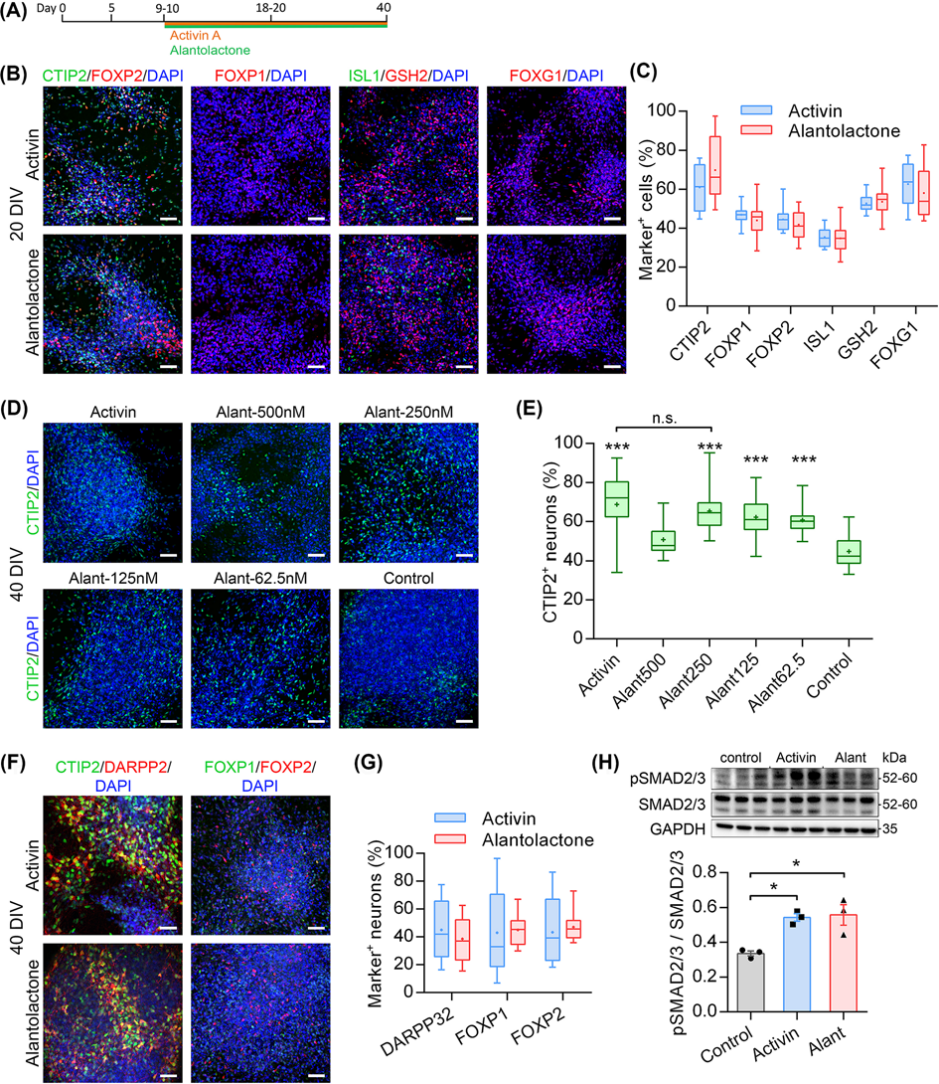
Efficient induction of MSN by TGFβ small molecule agonist (**A**) Schematic timeline of MSN differentiation. (**B** and **C**) Neuronal precursors expressing LGE and forebrain markers in Activin and Alantolactone (250 nM) cultures at 20DIV labelled and quantified for CTIP2, FOXP1, FOXP2, ISL1, GSH2, and FOXG1 (*n* = 20 per group). Box-and-whisker plots depict data for each condition; scale bars: 50 µm. (**D** and **E**) MSNs in Activin and varying concentrations of Alantolactone conditions at 40DIV labelled and quantified for CTIP2 (*n* = 40 per group; ***P<0.001 vs Control condition; n.s. = non-significant; one-way ANOVA with post-hoc Bonferroni). Box-and-whisker plots depict data for each condition; scale bar: 50 µm. (**F** and **G**) MSNs in Activin and Alantolactone (250 nM) cultures at 40DIV labelled and quantified for DARPP32, FOXP1 and FOXP2 (DARPP32: *n* = 10 per group; FOXP1/2: *n* = 30 per group). Box-and-whisker plots depict data for each condition; scale bars: 50 µm. (**H**) Phosphorylation of SMAD2/3 is greatly increased in Activin- and Alantolactone-treated cultures compared with basal control condition at 10DIV+5 (*n* = 3 per group; *P<0.05 vs Control condition; one-way ANOVA with post-hoc Bonferroni). Bar plots depict mean ± SEM for each condition with individual data points shown with markers.

Next, we investigated if Alantolactone, a small molecule agonist of TGFβ signalling, presents a suitable alternative to Activin to generate MSNs from hPSCs. Differentiating forebrain progenitors were treated with either Activin or Alantolactone (250nM) from 10DIV onwards (Figure 3A). At 20DIV, we observed a similar degree of LGE fate specification in both conditions as evidenced by comparatively high yields of cells expressing several well-established LGE and nascent MSN markers (Figure 3B). There were no significant differences between Activin and Alantolactone treatments across all markers analysed (Figure 3C).

To determine the most optimal Alantolactone concentration for generating MSNs, we quantified CTIP2^+^ neurons at 40DIV in five different conditions ranging from 0 to 500 nM Alantolactone and compared results to the basal control and Activin cultures (Figure 3D,E). The percentage of CTIP2^+^ neurons significantly increased in response to Alantolactone in a dose-dependent manner within a 0–250 nM range compared to control (Figure 3E). However, the number of CTIP2^+^ neurons at 500nM was similar to negative control levels, suggesting that too high activation of TGFβ pathway might be detrimental for MSN differentiation or survival. At 250nM, Alantolactone treatment yielded a similar number of CTIP2^+^ neurons compared with Activin (Figure 3E). Further experiment using the optimal concentration Alantolactone (250 nM) produced high percentages of DARPP32^+^, FOXP1^+^, and FOXP2^+^ neurons that were indistinguishable from our ‘gold-standard’ obtained by Activin (Figure 3F,G). We have tested LDN supplementation as in previous experiments, but it did not potentiate the effects of Alantolactone on MSN differentiation (data not shown).

To validate that Alantolactone can activate TGFβ pathway in neuronal precursors, we have analysed pSMAD2/3 levels in LGE-like progenitors at 10DIV+5. We observed a similar significant increase in pSMAD2/3 levels in both Activin and Alantolactone conditions compared with the basal control cultures (Figure 3H). Taken together, these results indicate that Alantolactone indeed offers an alternative solution to Activin to efficiently generate MSNs from hPSC-derived forebrain progenitors via SMAD2/3 pathway.

## Discussion

In the present study, we have begun to dissect the different components that could be contributing to the mechanism by which activation of TGFβ signalling induces LGE characteristics in hPSC-derived forebrain progenitors. Previous work using a zebrafish model of development demonstrated that the presence of Nodal, a TGF-β family member that shares some receptors with Activin, is necessary for Shh expression across the CNS and probably acts upstream of Shh to establish dorso-ventral patterning of the telencephalon [21]. Another group more recently showed that Smad3, an intracellular effector of TGF-β, Nodal and Activin signalling, is highly expressed in the subpallium throughout development, further highlighting the importance of these signalling pathways [22].

We have demonstrated that Activin exerts its effects via both ALK5 and ALK4, as dual ALK4/5 inhibitor was consistently more effective at inhibiting LGE marker up-regulation than ALK5 inhibitor alone. Furthermore, ALK4/5 inhibition in earlier experiments showed a complete abolishment of LGE marker expression, both at the mRNA and protein levels [11]. This is in agreement with the fact that Activin binds to both receptors *in vivo* and that they have the same intracellular effector proteins Smad2/3, which should have the same transcriptional targets [23].

Through the screening of multiple TGFβ family inhibitors, it was found that blocking BMP signalling at progenitor stage (10–20DIV) could enhance Activin-mediated induction of LGE marker gene expressions, leading to a change in the kinetics of the differentiation. Our findings suggest that blockade of BMP signalling accelerates MSN differentiation but does not increase the overall production of MSNs. BMPs are heavily involved in dorsal neural tube development but are specifically expressed in the dorsal midline of the forebrain [24]. It is therefore possible that the enhancement of Activin-induced LGE gene expression by BMP receptor inhibition is down to the suppression of a dorso-medial telencephalic fate, encouraging a more ventro-lateral patterning of the cells towards an LGE fate. As Activins and BMPs share some common ActR-II, it is also possible that LDN, which inhibits BMP Type I receptors ALK1/2/3/6, causes more Type II receptors to be available for Activin to bind and increase Smad2/3 phosphorylation [23].

We demonstrate for the first time that Alantolactone, a TGFβ small molecule agonist, offers a suitable alternative to Activin to efficiently generate MSNs from hPSC-derived forebrain progenitors. Previous research suggests that Alantolactone indirectly activates Smad2/3 signalling downstream of Activin by making ActR-II more bioavailable [17]. A recent study in zebrafish demonstrated that Alantolactone activated TGFβ signalling in a concentration-dependent manner to subsequently cause disruption of the circadian clock [25]. This is in line with our observations of similar Activin- and Alantolactone-induced increases in pSMAD2/3 levels and a dose-dependent effect of Alantolactone on efficiency of MSN differentiation. This novel finding provides further evidence that MSN fate is specified in forebrain progenitors via SMAD2/3 pathway activation.

In conclusion, we offer a fully defined and highly adaptable small molecule-based protocol via SMAD2/3 pathway to induce LGE fate in differentiating forebrain progenitors and to subsequently obtain high yield of MSNs. We provide extensive evidence to show that both Activin and Alantolactone, activators of TGFβ-SMAD2/3 signalling, efficiently generate MSNs from hPSCs. We also describe an option to further fine tune TGFβ activation in LGE progenitors by transiently inhibiting BMP signalling. This step accelerates MSN differentiation, which may prove to be useful for some hPSC lines that are slower in responding to differentiation cues or have intrinsically higher levels of BMP activity. Our study therefore establishes an essential role for TGFβ signalling in human MSN differentiation from PSCs.

## Summary

- Activin A and other TGFβ family members have been shown to exhibit a certain degree of promiscuity between their family of receptors.
- We previously developed an efficient differentiation protocol using Activin A to obtain medium spiny neurons (MSNs) from human pluripotent stem cells (hPSCs). However, the mechanism underlying Activin A-induced MSN fate specification remains largely unknown.
- Here, we begin to tease apart the different components of TGFβ pathways involved in MSN differentiation and demonstrate that Activin A acts exclusively via ALK4/5 receptors to induce MSN progenitor fate during differentiation. Moreover, we show that Alantolactone, an indirect activator of SMAD2/3 signalling, offers an alternative approach to differentiate hPSC-derived forebrain progenitors into MSNs.
- Further, fine tuning of TGFβ pathway by inhibiting BMP signalling with LDN193189 achieves accelerated MSN fate specification.
- The present study therefore establishes an essential role for TGFβ signalling in human MSN differentiation and provides a fully defined and highly adaptable small molecule-based protocol to obtain MSNs from hPSCs.

## Abbreviations

hPSC: human pluripotent stem cell
LGE: lateral ganglionic eminence
MSN: medium spiny neuron
